# Apoptotic Colitis in a Liver Transplant Recipient: A Case of Solid Organ Transplant-Associated Graft-Versus-Host Disease

**DOI:** 10.7759/cureus.108235

**Published:** 2026-05-04

**Authors:** Jong Woo Yoo, Amira Hamed, Ying-Hsia Chu, Ismail Elbaz Younes, Vanessa Humphreville, Punita Grover, Byoung Uk Park

**Affiliations:** 1 Department of Pathology, Yonsei University College of Medicine, Seoul, KOR; 2 Department of Laboratory Medicine and Pathology, University of Minnesota, Minneapolis, USA; 3 Department of Surgery, University of Minnesota, Minneapolis, USA; 4 Department of Medicine, University of Minnesota, Minneapolis, USA

**Keywords:** apoptotic colitis, donor-recipient chimerism, liver transplantation, post-transplant complication, solid organ transplant-associated gvhd

## Abstract

Solid organ transplant-associated graft-versus-host disease (SOT-GVHD) is a rare but often fatal complication following liver transplantation.

A 67-year-old man who underwent deceased donor liver transplantation subsequently developed progressive high-volume diarrhea and severe cytopenias with minimal initial cutaneous involvement. Colonic biopsy demonstrated apoptotic colitis with minimal lamina propria inflammation, suggestive of lower gastrointestinal (GI) GVHD. Donor-recipient chimerism analysis revealed significant donor-derived chimerism in peripheral blood and colonic tissue, confirming the diagnosis of SOT-GVHD. Maintenance immunosuppression was stopped to restore recipient immune function and eliminate donor-derived lymphoid cells; however, there was no clinical improvement. He was subsequently treated with high-dose systemic steroids, resulting in transient improvement. Despite this, he developed worsening diarrhea and pancytopenia, consistent with steroid-refractory GVHD, and passed away.

This case emphasizes the need for a high index of suspicion for SOT-GVHD in liver transplant recipients presenting with gastrointestinal symptoms and cytopenias, even in the absence of significant early cutaneous manifestations. Histopathology and donor chimerism analysis of the involved tissue can aid in early diagnosis. Initial therapeutic approaches vary significantly, both withdrawing immunosuppression to elicit a host-versus-graft response and augmenting immunosuppression with systemic steroids have been described. Regardless, SOT-GVHD is associated with a high rate of mortality. Novel approaches for early diagnosis, prevention, and treatment are needed.

## Introduction

Graft-versus-host disease (GVHD) is classically defined as a constellation of clinical manifestations caused by donor immunocompetent lymphoid cells after allogeneic hematopoietic stem cell transplantation (HCT). Although GVHD is primarily associated with HCT, it has also been reported following solid organ transplantation, a condition referred to as solid organ transplant-associated graft-versus-host disease (SOT-GVHD).

Among solid organ transplants, the incidence of GVHD is highest after intestinal transplantation, ranging from 5.6% to 19.2%, followed by liver transplantation, with an estimated incidence of 0.5% to 2%, with rare cases reported following kidney, lung, and heart transplantation [1,2]. The relatively high incidence observed in intestinal transplantation is thought to be related to the abundant lymphoid tissue within the intestinal graft. In contrast, GVHD after liver transplantation is exceedingly rare. 

Acute GVHD after HCT typically involves the skin, gastrointestinal tract, and liver. However, unlike acute GVHD following HCT, GVHD after liver transplantation predominantly affects the bone marrow, in addition to the skin and GI tract, while sparing the liver allograft itself [3]. Importantly, SOT-GVHD is diagnostically challenging, as its clinical and histologic features often overlap with more common post-transplant complications, including infection, drug toxicity, and acute rejection, which can delay recognition and contribute to its high mortality.

Here, we report a rare case of SOT-GVHD following liver transplantation, highlighting the diagnostic challenges and the role of histopathology and donor-recipient chimerism analysis in establishing the diagnosis.

## Case presentation

A 67-year-old man with end-stage liver disease secondary to metabolic-associated steatotic liver disease (MASLD) underwent deceased donor liver transplantation. Approximately 90 days post-transplant, he developed watery diarrhea (three to four episodes daily) accompanied by nausea, abdominal pain, generalized weakness, and weight loss. Computed tomography (CT) of the abdomen demonstrated ileal enteritis without colonic involvement. Stool PCR was positive for norovirus. He received supportive care for presumed infectious enteritis and was discharged; however, high-volume watery diarrhea persisted (1-2 L/day), prompting readmission one week later with fever, hypotension, acute kidney injury, poor oral intake, and ongoing diarrhea.

**Figure 1:**
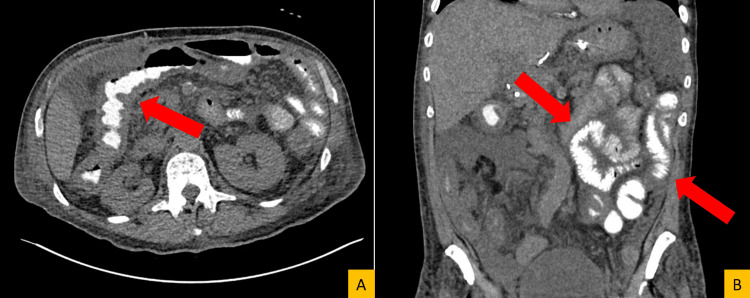
CT findings of small and large bowel wall thickening with mesenteric edema and ascites in a post-liver transplant patient. Axial (A) and coronal (B) CT images of the abdomen and pelvis, performed with oral contrast only, demonstrate diffuse circumferential wall thickening involving both small and large bowel loops. The abnormalities are most pronounced in distal small bowel and colonic segments. Associated mesenteric stranding and moderate-volume ascites are present. Selected bowel loops are highlighted for illustration. No discrete intra-abdominal abscess or focal collection is identified, although evaluation is limited by the absence of intravenous contrast.

On admission, he appeared cachectic and chronically ill. Laboratory evaluation demonstrated leukopenia with a white blood cell count of 3.7 × 10³/µL and absolute lymphopenia, severe anemia with hemoglobin 6.6 g/dL, and a platelet count of 170 × 10³/µL. Repeat CT imaging demonstrated diffuse bowel wall thickening involving both the small and large intestine, most pronounced in the ileum and colon (Figure 1). An extensive infectious evaluation - including blood and urine cultures, respiratory viral testing, stool testing for Clostridioides difficile and other enteric pathogens, and targeted viral and parasitic studies (including cytomegalovirus, herpes simplex virus, adenovirus, and microsporidia) - was repeatedly negative. Mycophenolate was discontinued and switched to azathioprine, given concern for drug-induced diarrhea. Multiple anti-diarrheal agents were trialed without benefit. Pancreatic insufficiency was considered but deemed unlikely. Diarrhea progressed to 2-3 L/day, complicated by ileus, necessitating further evaluation for GVHD.

The hospital course was complicated by acute kidney failure due to GI losses, requiring intermittent hemodialysis, recurrent episodes of fever and hypotension likely due to gut bacterial translocation, and toxic metabolic encephalopathy.

**Figure 2:**
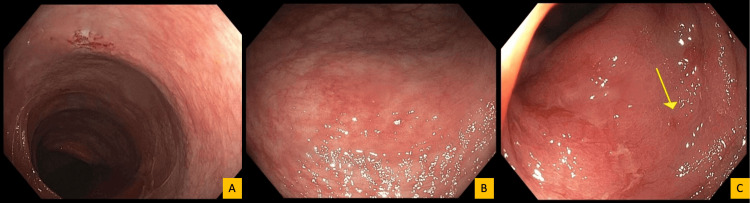
Colonoscopy findings; patchy, subtle mucosal abnormalities are demonstrated across multiple colonic segments. (A) Ascending colon showing mildly erythematous (hyperemic) mucosa without discrete ulceration, mass lesion, or active bleeding. (B) Descending colon demonstrating patchy, mildly erythematous mucosa, similar to changes identified in the transverse colon and hepatic flexure, where the erythema was slightly more prominent but overall subtle. (C) Sigmoid colon showing a few localized, non-bleeding, subtle erosions (arrow), without stigmata of recent bleeding. Targeted biopsies were obtained from involved colonic segments using cold forceps for histologic evaluation.

Colonoscopy demonstrated diffusely abnormal but subtle colonic mucosal changes characterized by edema, friability, and patchy erythema without discrete ulceration or mass lesions (Figure 2). A few localized, non-bleeding erosions were identified in the sigmoid colon. Targeted biopsies were obtained from multiple colonic segments.

**Figure 3:**
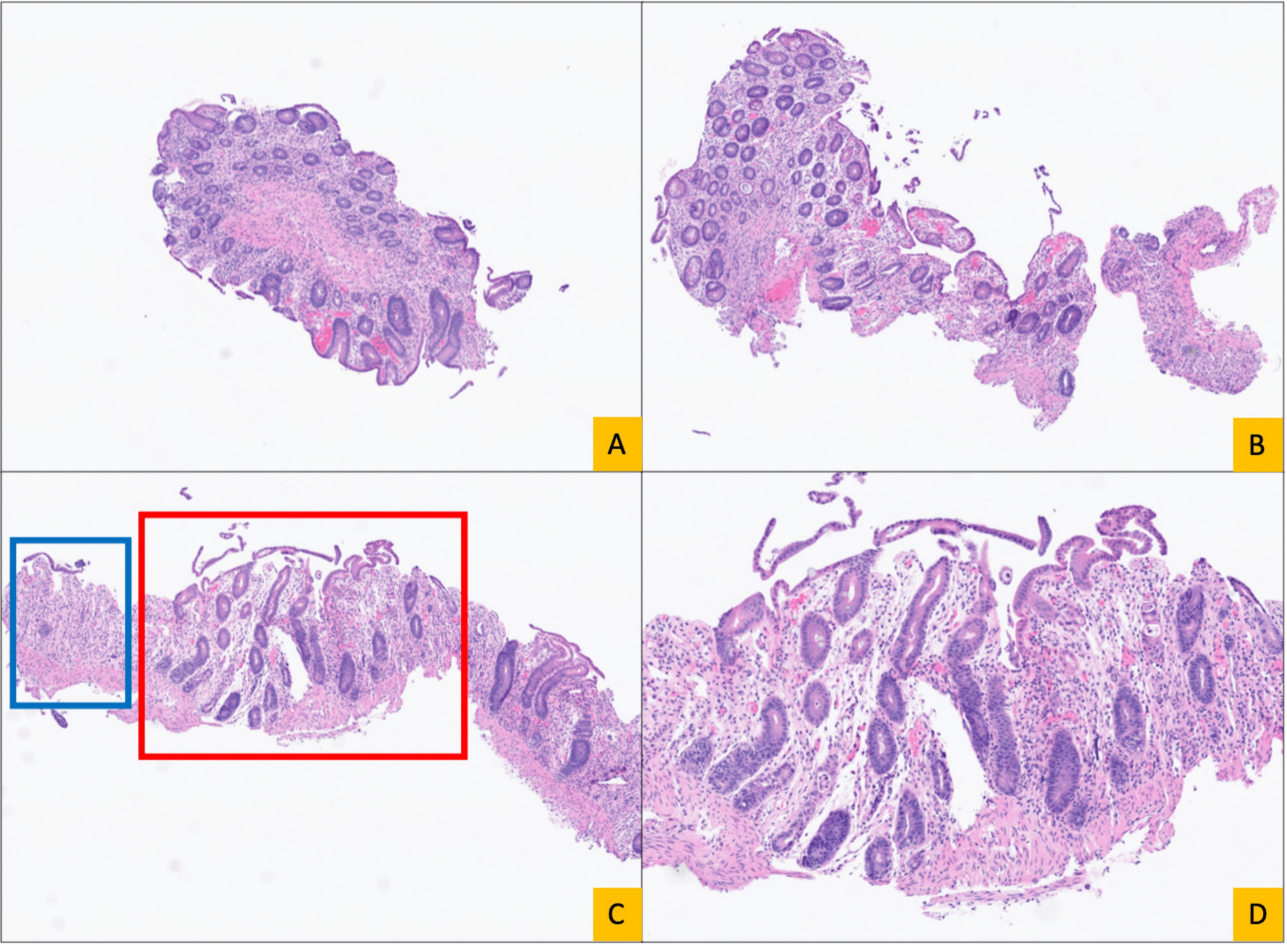
Histologic findings of colonic biopsies demonstrating an apoptotic colitis/colopathy pattern, low magnification. Hematoxylin and eosin-stained sections from multiple colonic biopsy sites demonstrate a diffuse pattern of epithelial injury at low magnification, characterized by crypt dropout, architectural disarray, and superficial erosions. (A) Right colon biopsy (5×) showing areas of crypt loss and mucosal injury. (B) Left colon biopsy (5×) demonstrating similar architectural injury with crypt dropout and superficial erosion. (C) Hepatic flexure biopsy from an erythematous area (5×) showing prominent epithelial injury with crypt dropout (blue box). (D) Intermediate magnification view (10×) of the region of interest panel C (red box), highlighting epithelial injury.

**Figure 4:**
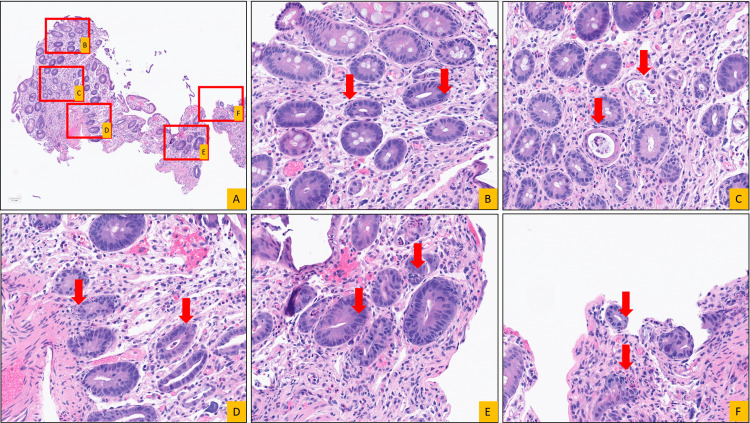
High-magnification histologic features of apoptotic epithelial injury in colonic biopsies. Hematoxylin and eosin-stained sections from a colonic biopsy demonstrate an apoptotic colitis/colopathy pattern. (A) Low magnification (8×) highlights patchy epithelial injury with crypt attenuation/dropout and focal surface epithelial denudation; red boxes denote areas shown at higher magnification. (B-F) High-power views (40×) of the boxed areas show numerous apoptotic bodies within crypt epithelium (arrows), with associated epithelial injury disproportionate to the accompanying lamina propria inflammation. Neutrophilic activity is limited (rare intraepithelial neutrophils), while eosinophils are readily identified. No granulomas or dysplasia are identified.

Histologic examination of the colonic biopsies demonstrated epithelial injury characterized by prominent crypt epithelial apoptosis with associated crypt dropout, epithelial denudation, and superficial erosions, accompanied by relatively minimal inflammatory infiltrate, consistent with an apoptotic colitis/colopathy pattern (Figures 3, 4). No granulomas or dysplasia were identified. Although this pattern of injury is non-specific, in the post-solid organ transplant setting, the differential diagnosis includes drug-related injury, viral infection, and graft-versus-host disease. Immunohistochemical stains for cytomegalovirus, herpes simplex virus, and adenovirus were negative.

Taken together, the combination of persistent high-volume diarrhea refractory to standard management, negative infectious work-up, progressive cytopenias, subtle but diffuse mucosal abnormalities on endoscopy, and an apoptotic colitis pattern on histology raised strong concern for SOT-GVHD.

**Figure 5:**
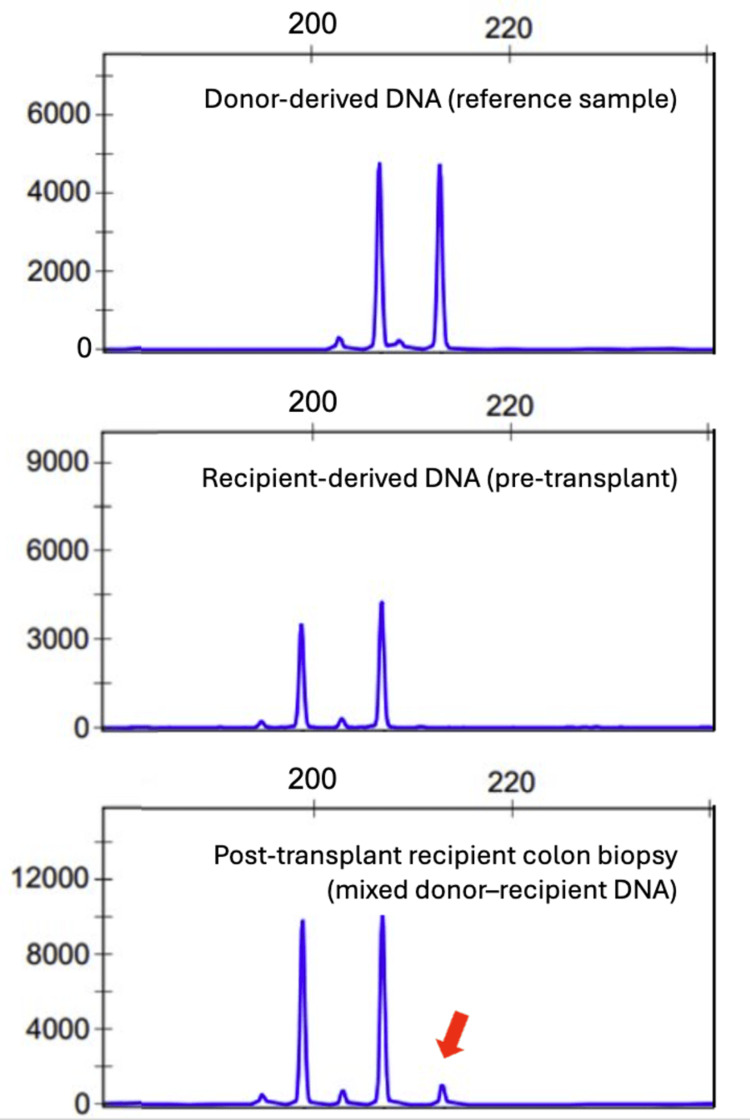
Chimerism studies revealed donor alleles in the sigmoid colon biopsy. The donor was heterozygous for two distinct alleles, with amplicon sizes of 207 and 213 base pairs (bp) for the D21S11 marker (upper panel). The recipient’s pretransplant sample demonstrated two alleles at 199 and 207 bp (middle panel). In the post-transplant colon biopsy (lower panel), 11% donor-derived DNA was detected, as evidenced by a minor peak at 213 bp (arrow).

Given high clinical suspicion for GVHD, DNA chimerism was analyzed by polymerase chain reaction (PCR) amplification of 16 short tandem repeat (STR) polymorphic genetic markers (TH01, CSF1PO, D16S539, D2S1338, D3S1358, vWA, FGA, Amelogenin, D8S1179, D21S11, D18S51, D5S818, D13S317, D19S433, TPOX, and D7S820). DNA extracted from the sigmoid colon biopsy specimen demonstrated 11% donor-derived and 89% recipient, confirming the lower GI GVHD (Figure 5). The peripheral blood was fractionated using the EasySep™ magnetic cell separation technology (STEMCELL Technologies, Vancouver, Canada) to isolate CD3-positive T cells and CD33/66b-positive myeloid cells for lineage-specific chimerism testing. This demonstrated a substantial population of circulating donor-derived T lymphocytes, with approximately 12% donor cells in the CD3-positive fraction, while the CD33/66b-positive myeloid fraction showed no detectable donor contribution.

Bone marrow biopsy demonstrated normocellular marrow (approximately 30-40%), myeloid-predominant trilineage hematopoiesis with relative eosinophilia. There was no evidence of malignancy or dysplasia. Chimerism testing on the bone marrow showed 100% recipient DNA. Notably, there was no marrow hypoplasia or aplasia, and no evidence of donor-derived hematopoietic engraftment, arguing against suggesting that bone marrow involvement by GVHD.

Initial management was the withdrawal of maintenance immunosuppression. Azathioprine and cyclosporin were stopped around day 118 post-transplant and replaced with prednisone 10 mg daily. Over the subsequent two weeks, GVHD progressed, with stage 3 skin involvement (>50% body surface area), persistent stage 3 lower GI GVHD (>1.5 L of diarrhea per day), and an expansion of circulating donor CD3-positive lymphocytes to 37%. High-dose systemic steroids were initiated (methylprednisolone 48 mg/m2) per institutional acute GVHD protocol. Given the severity of GVHD, pulse methylprednisolone (1000 mg every three days for three doses) was added. This resulted in notable clinical improvement with a partial GVHD response. After one week of steroids, skin GVHD was resolved with improvement of lower GI GVHD to stage 2 (1-1.5 L diarrhea per day).

Due to persistent GI GVHD, ruxolitinib 5 mg twice daily was initiated. Within three days, the patient developed severe pancytopenia (WBC 0.26 × 10³/µL, Hb 8.4 g/dL, platelets 34 × 10³/µL), attributable either to evolving marrow GVHD or ruxolitinib toxicity. Given the multiorgan dysfunction, profound debility, and limited therapeutic options, his care was transitioned to a more comfort-focused approach. He died on day 149 post-transplant.

## Discussion

Despite its name, SOT-GVHD differs substantially from classic acute GVHD following HCT, beginning with its underlying pathophysiology. In classic acute GVHD following HCT, recipient bone marrow is ablated to allow donor hematopoietic stem cells engraftment. The donor-derived immune system recognizes the recipient as foreign, leading to immune-mediated injury to recipient tissues characteristic of GVHD.

In contrast, after solid organ transplantation, the recipient’s bone marrow and immune system remain intact. Under usual circumstances, recipient immune cells attack transplanted organs, including donor-derived lymphoid cells, preventing their activation and expansion. However, immunosuppressive therapy used to prevent allograft rejection suppresses this protective immune surveillance, allowing donor lymphoid cells to survive, proliferate, and potentially induce SOT-GVHD [4].

SOT-GVHD is a highly lethal condition, and early diagnosis and prompt treatment are critical [5]. Clinically, SOT-GVHD closely resembles classic acute GVHD, with common manifestations including fever, skin rash, and diarrhea. When the native liver is present, hepatic involvement is also frequently observed. Bone marrow involvement, manifesting as marrow aplasia or hypoplasia, leukopenia or pancytopenia, is particularly characteristic of SOT-GVHD and is associated with a poor prognosis [3]. Diagnosis is frequently delayed due to the non-specific nature of presenting symptoms and their overlap with more common complications like drug toxicities and infections. Accordingly, when these clinical features develop following solid organ transplantation, GVHD should be strongly suspected, and further evaluation with biopsy of the affected organs and peripheral blood chimerism testing should be pursued.

The definitive diagnosis of GVHD requires histopathologic confirmation by biopsy of affected organs. As in SOT-GVHD following liver transplantation, the skin, gastrointestinal tract, and bone marrow are the most involved sites, and biopsies are typically obtained from these organs [4]. Among them, skin biopsy is often preferred due to its relatively low invasiveness.

Histopathologic features of SOT-GVHD are similar to those of classic GVHD. In the gastrointestinal tract, the hallmark finding is apoptotic colitis. The National Institutes of Health (NIH) guidelines recommend that at least one apoptotic body be present per biopsy fragment for the diagnosis of GVHD in colonic biopsies [6]. In contrast, many pathologists use a stricter cutoff of six apoptotic bodies per 10 contiguous crypts [6]. This discrepancy reflects the inherent trade-off between reducing false-positive diagnoses and increasing diagnostic sensitivity. Additional histologic features include crypt injury with apoptotic crypt abscesses and naked neuroendocrine cell clusters [7].

Although histologic grading systems for gastrointestinal GVHD have been proposed, their correlation with clinical staging and disease severity remains limited, and significant interobserver variability exists [6]. Accordingly, clinicopathologic correlation is essential, with histologic findings interpreted in conjunction with the clinical presentation and disease course rather than in isolation.

Importantly, apoptotic colitis is not specific to GVHD and may also be observed in drug-induced injury and infectious colitis. In this case, the differential diagnosis was approached in a stepwise manner. Infectious etiologies were first considered, particularly cytomegalovirus (CMV), which is a common cause of colitis in transplant recipients. Although viral inclusions may be identified on routine histology, immunohistochemistry is frequently used to aid evaluation of CMV colitis and is routinely performed in transplant settings [8]; in this case, infectious studies, including CMV immunohistochemistry, were negative.

Drug-induced injury was then evaluated, with mycophenolate mofetil (MMF)-induced colitis representing a key consideration given its widespread use and histologic overlap with GVHD. Histologic findings that support MMF-induced colitis over GVHD include increased lamina propria eosinophils (often exceeding 15 eosinophils per 10 high-power fields) and a relative absence of apoptotic microabscesses. In contrast, the presence of apoptotic microabscesses and endocrine cell aggregates, which are thought to reflect relative preservation of endocrine cells amid selective injury to the non-endocrine crypt epithelium, favors GVHD [9].

In our case, the patient presented with gastrointestinal symptoms, including diarrhea, nausea, and abdominal pain. Colonoscopy with biopsy was performed to evaluate for GVHD and demonstrated apoptotic colitis with prominent epithelial injury and crypt dropout, with minimal inflammatory infiltrate in the lamina propria. These findings were consistent with GVHD; infectious etiologies were excluded by immunohistochemistry, and MMF had been discontinued at least two weeks before biopsy. In addition, donor chimerism testing revealed donor-derived cells comprising 37% of peripheral blood leukocytes and 10% of cells in the colonic biopsy specimen, further confirming the diagnosis of SOT-GVHD.

Assessment of donor chimerism is valuable for diagnosing SOT-GVHD. Previous studies have described donor chimerism as a marker for both graft rejection and GVHD risk. Elevated donor chimerism reflects an increased proportion of donor-derived lymphoid cells, indicating active donor immune cell expansion, which is suggestive of GVHD when correlated with compatible clinical features. Chimerism testing is most commonly performed by PCR amplification of polymorphic STR markers followed by electrophoresis separation of distinct alleles. Although cut-off values vary depending on the transplanted organ, donor chimerism, particularly T-cell chimerism, exceeding 1% is generally considered clinically significant in liver transplantation [3,10]. Chimerism analysis can be performed using either peripheral blood or tissue biopsy specimens, with comparable diagnostic thresholds. Nevertheless, chimerism results must be interpreted with caution, as donor chimerism may be transiently increased during the early post-transplant period [11].

Currently, there is no established standard treatment for SOT-GVHD. Corticosteroids are generally considered first-line therapy, largely based on treatment strategies for classic acute GVHD, although robust evidence specific to SOT-GVHD is lacking. The typical initial dose is prednisone 1-2 mg/kg/day or equivalent. With steroid-responsive GVHD, steroids are tapered over 10 weeks. In cases of steroid-refractory disease (no improvement after four days or progression after seven days of high-dose systemic steroids), second-line agents should be considered. Ruxolitinib, anti-thymocyte globulin, interleukin-2 receptor antagonists, and tumor necrosis factor alpha inhibitors have been used with variable success [3]. Intensifying immunosuppression increases the risk of infections; antiviral, anti-fungal, and *Pneumocystis jirovecii* pneumonia prophylaxis is recommended. In cases of marrow involvement by GVHD with severe pancytopenia, allogeneic stem cell transplant can be considered.

An alternative therapeutic approach suggested by Kneifel et al. involves reducing or discontinuing immunosuppressive agents [4]. This strategy is based on the preserved recipient immune system in SOT-GVHD, which may regain the capacity to eliminate donor lymphoid cells once immunosuppression is minimized. Moreover, infection remains the most common cause of death in patients with GVHD, which further supports consideration of immunosuppressive reduction [12]. However, this strategy carries the risk of initial worsening of GVHD symptoms and has limited prospective data. Therefore, it may be most appropriate in earlier stages of GVHD without significant visceral organ involvement.

In our case, immunosuppressive therapy was initially minimized; however, clinical deterioration necessitated escalation to high-dose corticosteroid therapy. Although a partial clinical response was achieved, the patient’s clinical course remained complicated by significant debility, malnutrition, and multiorgan dysfunction.

## Conclusions

We present a rare case of solid organ transplant-associated graft-versus-host disease following liver transplantation. While based on a single case, these findings underscore the importance of maintaining a high index of suspicion for GVHD in liver transplant recipients presenting with unexplained gastrointestinal symptoms, even in the absence of cutaneous manifestations. Diagnosis in this case was established through histopathologic evaluation demonstrating apoptotic colitis, with exclusion of infectious and drug-related etiologies, and supported by donor chimerism analysis. Despite appropriate diagnostic evaluation, outcomes in SOT-GVHD remain poor, highlighting the need for improved diagnostic strategies and therapeutic approaches.

## References

[1] Vianna R, Farag A, Gaynor JJ (2020). Association of alemtuzumab induction with a significantly lower incidence of GVHD following intestinal transplantation: results of 445 consecutive cases from a single center. Transplantation.

[2] Smith DM, Agura E, Netto G (2003). Liver transplant-associated graft-versus-host disease. Transplantation.

[3] Murali AR, Chandra S, Stewart Z, Blazar BR, Farooq U, Ince MN, Dunkelberg J (2016). Graft versus host disease after liver transplantation in adults: a case series, review of literature, and an approach to management. Transplantation.

[4] Kneifel F, Vogel T, Bormann E (2023). Graft-versus-host disease following liver transplantation: a systematic review of literature. Hepatol Commun.

[5] Obana A, Akabane M, Mumtaz K (2025). Graft-versus-host disease after liver transplantation: a global review of pathogenesis, diagnosis, and treatment strategies. Transplant Rev (Orlando).

[6] Park BU, Ahmed F, Hartley CP (2026). Histopathologic evaluation of gastrointestinal graft-versus-host disease: opportunities for improvement based on a survey of practicing pathologists. Ann Diagn Pathol.

[7] Washington K, Jagasia M (2009). Pathology of graft-versus-host disease in the gastrointestinal tract. Hum Pathol.

[8] Karamchandani DM, Chetty R (2018). Apoptotic colopathy: a pragmatic approach to diagnosis. J Clin Pathol.

[9] Lampert IA, Thorpe P, van Noorden S, Marsh J, Goldman JM, Gordon-Smith EC, Evans DJ (1985). Selective sparing of enterochromaffin cells in graft versus host disease affecting the colonic mucosa. Histopathology.

[10] Fu J, Zuber J, Shonts B (2021). Lymphohematopoietic graft-versus-host responses promote mixed chimerism in patients receiving intestinal transplantation. J Clin Invest.

[11] Domiati-Saad R, Klintmalm GB, Netto G, Agura ED, Chinnakotla S, Smith DM (2005). Acute graft versus host disease after liver transplantation: patterns of lymphocyte chimerism. Am J Transplant.

[12] Chinnakotla S, Smith DM, Domiati-Saad R (2007). Acute graft-versus-host disease after liver transplantation: role of withdrawal of immunosuppression in therapeutic management. Liver Transpl.

